# The Effect of Resting Heart Rate on the New Onset of Microalbuminuria in Patients With Type 2 Diabetes: A Subanalysis of the ROADMAP Study

**DOI:** 10.1097/MD.0000000000003122

**Published:** 2016-04-18

**Authors:** Roland E. Schmieder, Peter Bramlage, Hermann Haller, Luis M. Ruilope, Michael Böhm

**Affiliations:** From the Department of Nephrology and Hypertension (RES), University Hospital, Erlangen; Institute for Pharmacology and Preventive Medicine, (PB), Mahlow; Department of Nephrology and Hypertension, Hannover Medical School (HH), Hannover; Institute of Investigation and Hypertension Unit (LMR), Hospital 12 de Octubre, Madrid, Spain; and Department for Internal Medicine III (MB), Saarland University Medical Center, Homburg, Germany.

## Abstract

The association between resting heart rate and new-onset microalbuminuria in patients with type 2 diabetes is not clear. The objective of the current analysis was to assess the relationship between heart rate and incidence of microalbuminuria in patients with type 2 diabetes.

Data from the Randomised Olmesartan and Diabetes Microalbuminuria Prevention (ROADMAP) study were retrospectively analyzed. New-onset microalbuminuria was documented and related to heart rate as recorded at baseline and last assessment, and the mean of the measurements taken during the double-blind part of the ROADMAP trial.

Patients (n = 4299) had a mean age of 57.8 ± 8.7 years and 46.3% were male. Characteristics were not different between the olmesartan and the placebo groups, except for a higher systolic blood pressure (136.7 vs 135.7 mm Hg; *P* = 0.04) and albumin creatinine ratio (5.9 vs 5.5; *P* = 0.03).

Increased risk of microalbuminuria was found with increasing heart rate, independent of whether baseline [highest vs lowest quartile odds ratio (OR) 1.39; 95% confidence interval (95% CI) 1.03–1.87; *P* = 0.032], last assessment (OR 1.71; 95% CI 1.26–2.31; *P* = 0.001), or mean heart rate was considered (OR: 1.77; 95% CI: 1.30–2.41; *P* = 0.0003). The greater risk of new-onset microalbuminuria with a high baseline heart rate was also found when data were adjusted for mean systolic blood pressure (OR: 1.35; 95% CI: 1.00–1.82; *P* = 0.0496; interaction *P* < 0.0001). Although there was no risk increase with baseline heart rate in the placebo group (*P* = 0.8253 for trend), microalbuminuria was less frequent in patients receiving olmesartan in the low heart rate quartiles (*P* = 0.002 for trend).

A low heart rate reduces the risk of patients with type 2 diabetes developing microalbuminuria, independent of blood pressure. The data demonstrate potential benefits of reducing the heart rate of type 2 diabetes patients, and indicate that olmesartan could, in particular, reduce the risk of microalbuminuria in patients with low heart rate.

## INTRODUCTION

The association between resting heart rate (HR) and mortality has been observed in a broad spectrum of patients,^1,2^ including large clinical^3,4^ and epidemiologic cohorts of healthy individuals,^5,6^ and those with hypertension, coronary artery disease, and left ventricular systolic dysfunction.^7–10^ This association is independent of traditional cardiac risk factors and may assist in explaining the individual risk unaccounted for in other models. Microalbuminuria (MAU) is indicative of increased vascular permeability and has been associated with a greater risk for cardio-reno-vascular morbidity and mortality.^11,12^ The condition has been reported to develop at a rate of about 2% of patients per year after initial type 2 diabetes diagnosis.^13^

A raised HR has been found to be an independent predictor for the development of MAU in hypertensive patients with high cardiovascular risk.^14,15^ It has been shown that anti-hypertensive therapies are able to reduce the incidence of MAU, with blockers of the renin-angiotensin system being particularly effective.^16,17^ Furthermore, it was shown that a pharmacologically induced reduction in HR resulted in improvements of endothelial function in mice,^18^ and thus has the potential to reduce new-onset MAU in humans. The mechanism by which HR affects the occurrence of MAU in diabetic patients remains unclear, and it is likely that a combination of factors contribute to its development. Increases in pulse waves, glomerular pressure, and basement membrane permeability; inflammatory effects; and pro-atherosclerotic activity have all been postulated to be involved in the process.^14,15,19^

In order to clarify the effects of HR on new-onset MAU in patients with type 2 diabetes, and the potential effects of blocking the renin-angiotensin system, we analyzed data from the Randomised Olmesartan and Diabetes Microalbuminuria Prevention (ROADMAP) study.^16,20,21^ This is one of the largest datasets so far that has been used to assess the associations between resting HR and new-onset MAU in patients at a high vascular risk.

## METHODS

### Study Design and Patients

The ROADMAP study was a randomized, placebo-controlled, double-blind, multinational trial designed with the aim of determining the effect of the angiotensin receptor blocker (ARB) olmesartan on the onset of MAU in patients with type 2 diabetes.^20,21^ Patients were included if, in addition to having type 2 diabetes, they had 1 of the following cardiovascular risk factors: total cholesterol >200 mg/dL, high-density lipoprotein (HDL) cholesterol <40 mg/dL, triglycerides >150 and <400 mg/dL, systolic blood pressure (SBP) ≥130 mm Hg and diastolic blood pressure (DBP) ≥80 mm Hg, body mass index (BMI) ≥28 kg/m^2^, waist circumference >102 cm (men) or >88 cm (women), or smoking more than 5 cigarettes a day. Furthermore, they were required to have normoalbuminuria at screening, defined as ≤25 mg of albumin per g of urine creatinine (men) or ≤35 mg/g (women). Patients were excluded if they suffered from severe hyperlipidaemia, had documented renal and/or reno-vascular disease, had experienced a myocardial infarction (MI) or stroke within the previous 6 months, had a history of drug or alcohol abuse, had a known allergy or lack of response to angiotensin II-antagonists, or were receiving an ARB or angiotensin-converting enzyme (ACE)-inhibitor. A full list of inclusion and exclusion criteria is available in the previously published study protocol.^21^ Patients were randomized to placebo or to 40 mg olmesartan medoxomil (oral, once daily) and treated until 325 cases of new-onset MAU (primary endpoint) had been recorded. This resulted in a median follow-up of 3.2 years.

The study was carried out in accordance with the Declaration of Helsinki and its amendments. The ethics committee at each participating center approved the study, and written informed consent was obtained from each patient.

### Documentation

For the present subanalysis, resting HR was documented at baseline and at the end of the double-blind period of the trial. Furthermore, HR measurements were taken at intervals during this period (week 4, week 12, month 6, and then every 6 months).

The presence of MAU was determined by a morning spot urine test performed at baseline, week 12, month 6, and every 6 months thereafter. It was defined as >25 mg of albumin per g of urine creatinine for men or >35 mg/g for women. Any single elevation in the urinary albumin-to-creatinine ratio (UACR) required confirmation by at least 1 additional positive result out of 2 further tests within the subsequent 2 weeks.

For the determination of an association of HR with MAU, HR at baseline, HR at the last follow-up in the database, and mean HR calculated from all measurements taken from baseline to the end of the double-blind period of the study were used.

### Statistics

Differences in patient characteristics between the olmesartan and placebo arms were determined using a *t* test (continuous variables) or the Chi-squared test (categorical variables). The total population of patients (treatment and placebo arms) was divided into quartiles according to HR at baseline, with the lowest quartile used as the reference. Trends between quartiles were calculated using the Cochran–Armitage trend test. Odds ratios and corresponding 95% confidence intervals (CIs) were calculated using logistic regression. Data are presented as unadjusted, and adjusted for the covariates age, gender, obesity, mean SBP, treatment assignment, beta-blocker use, and calcium channel blocker use. Data were analyzed using the SAS version 9.3 (SAS Institute Inc., Cary, NC).

## RESULTS

### Patient Population

A total of 4447 patients were included in the ROADMAP study and were followed for a mean duration of 3.2 years. For the analysis of the association of HR with new-onset MAU, data from 4299 patients were available at baseline (Table 1). Patients had a mean age of 57.8 ± 8.7 years and 46.3% were male. The mean BMI was 31.0 kg/m^2^, with over 50% of patients having a BMI > 30 kg/m^2^. In terms of blood pressure, mean SBP was 136.2 ± 15.2 mm Hg and mean DBP was 80.5 ± 9.5 mm Hg. There were no apparent differences between the olmesartan and placebo groups for any of the aforementioned characteristics apart from SBP (*P* = 0.04).

**TABLE 1:**
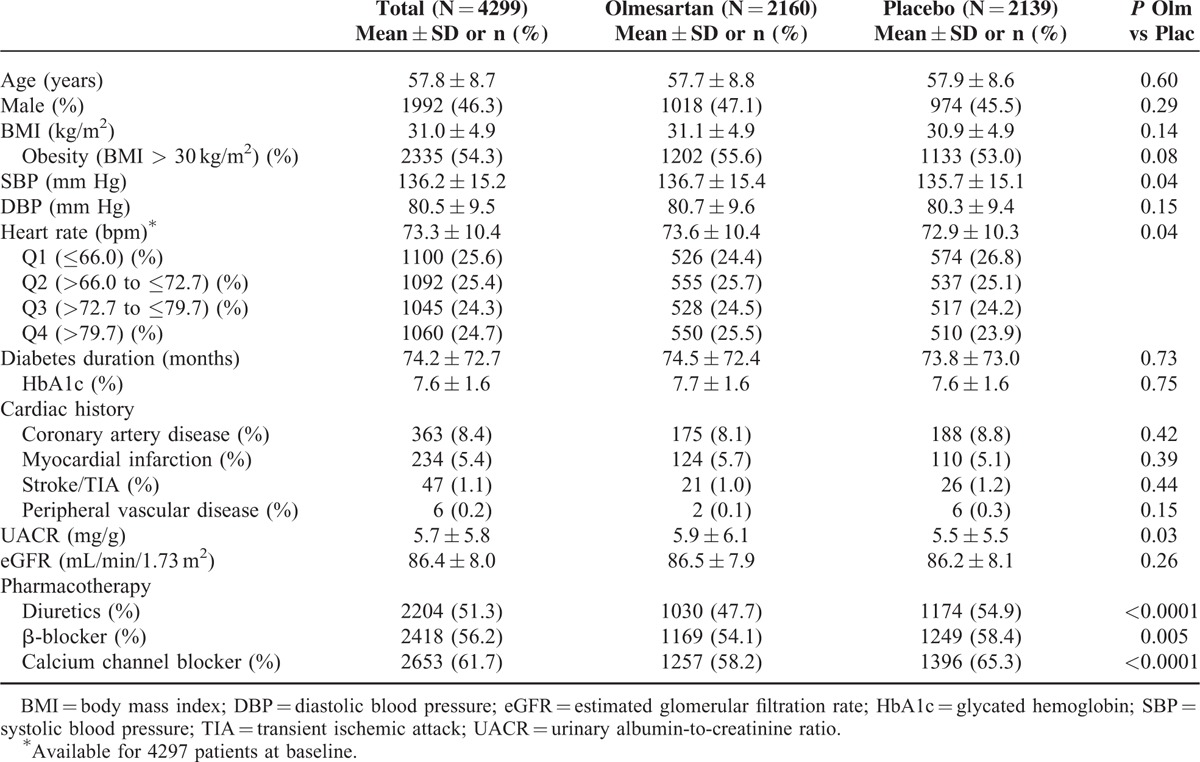
Patient Characteristics at Baseline

The patients had a mean diabetes duration of 74.2 ± 72.7 months, with a mean HbA1c level of 7.6 ± 1.6%. With regard to cardiac history, 363 (8.4%) patients suffered from coronary artery disease, while 234 (5.4%) had experienced an MI. The mean UACR was 5.7 ± 5.8 mg/g and the eGFR was 86.4 ± 8.0 mL/min/1.73 m^2^. UACR at baseline was slightly higher in the olmesartan than placebo groups (5.9 ± 6.1 vs. 5.5 ± 5.5; *P* = 0.03).

Patients were divided into quartiles according to HR at baseline, HR at last assessment, and according to the mean calculated from all measurements taken between baseline and the end of the double-blind period of the ROADMAP study. These quartiles, and the proportions of patients in each, are defined in eTable 1, wherein it can be seen that the HR values that correspond to the baseline and last assessment quartiles are almost identical. This shows that HR remained fairly constant during the study period.

### Frequency of Microalbuminuria According to Heart Rate (Crude)

**FIGURE 1:**
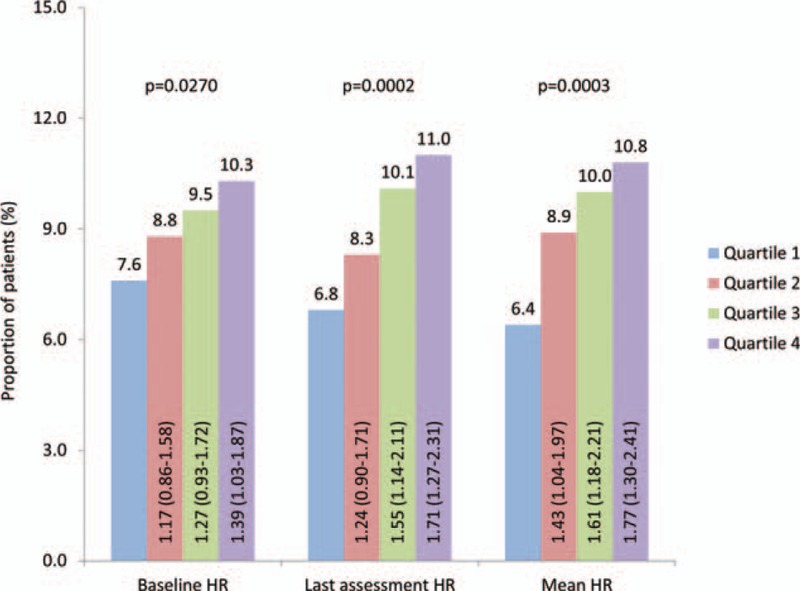
Frequency of new-onset microalbuminuria according to heart rate. Odds ratios (OR) with 95% confidence intervals (CI). Columns compare Quartiles 2, 3, and 4 with Quartile 1. *P* values derived from a Cochran–Armitage test for trend.

An increased risk of MAU was found with increasing HR, independent of whether baseline HR, last assessment HR, or mean HR was considered (Figure 1). In a paired comparison, patients from higher baseline HR quartiles showed a greater risk of MAU than MAU rates observed in the lowest baseline HR quartiles (OR 1.39; 95% CI 1.03–1.87; *P* = 0.032). When the same comparison was carried out using the HR measured at the last assessment, patients in the highest HR quartile again had a greater risk of MAU in comparison to those in the lowest HR quartile (OR 1.71; 95% CI 1.26–2.31; *P* = 0.001). Furthermore, patients in the third HR quartile also had a greater risk of MAU in comparison to those in the lowest HR quartile when the last assessment measurements were evaluated (OR: 1.55; 95% CI: 1.14–2.11; *P* = 0.005).

When using the mean HR to divide the patients into quartiles, those in the highest HR quartile had a greater risk of MAU in comparison to those in the lowest (OR: 1.77; 95% CI: 1.30–2.41; *P* = 0.0003), as did the third (OR: 1.61; 95% CI: 1.18–2.21; *P* = 0.003), and the second (OR: 1.43; 95% CI: 1.04–1.97; *P* = 0.03) HR quartiles.

### Frequency of Microalbuminuria According to Heart Rate (Covariate Adjusted)

**FIGURE 2:**
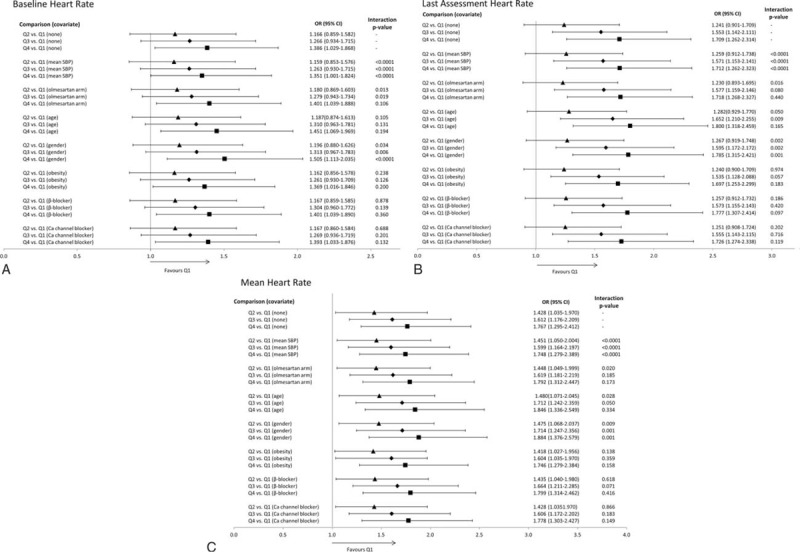
Risk of new-onset microalbuminuria according to baseline heart rate quartiles. Covariate adjusted risk of new-onset microalbuminuria according to (A) baseline HR, (B) last assessment HR, and (C) mean HR. SBP = systolic blood pressure. Triangles, Q2 vs Q1; diamonds, Q3 vs Q1; squares, Q4 vs Q1.

The greater risk of new-onset MAU found for the highest baseline HR quartile in comparison to the lowest was also found when the data were adjusted for mean SBP (OR: 1.35; 95% CI: 1.00–1.82; *P* = 0.0496), with the interaction with this covariate found to be significant (interaction *P* < 0.0001; Figure 2A). Furthermore, the interaction with SBP was also found to be significant when comparing the lowest quartile with the second and third baseline HR quartiles (interaction *P* < 0.0001 for both). The difference between the lowest and highest baseline HR quartiles was also evident when the data were adjusted for olmesartan treatment, age, gender, obesity, β-blocker treatment, or calcium channel blocker treatment. However, the interactions with these covariates were not found to be significant (interaction *P* > 0.10), with the exception of gender, which displayed interactions when comparing the lowest HR quartile with the highest (interaction *P* < 0.0001) and the third (*P* = 0.006).

When HR at last assessment or mean HR was used to define quartiles, mean SBP was the only covariate to show a significant interaction with the risk of new-onset MAU (interaction *P* < 0.0001 for comparisons of lowest HR quartile with second, third, and highest; Figure 2B C). Moderate interactions were seen for gender and age.

### Risk of Microalbuminuria According to Heart Rate and Treatment Assignment

**FIGURE 3:**
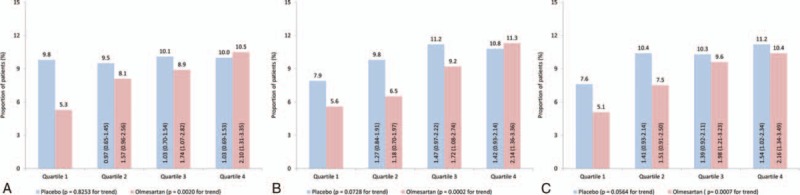
Frequency of new-onset microalbuminuria according to heart rate by treatment group. Frequency of new onset MAU during double-blind period according to (A) baseline HR, (B) last assessment HR, and (C) mean HR, in the placebo and olmesartan groups. Odds ratios (OR) with 95% confidence intervals (CI). Columns compare Quartiles 2, 3, and 4 with Quartile 1 within the placebo or olmesartan groups. *P* values derived from a Cochran–Armitage test for trend.

When patients were categorized according to baseline HR, there were no clear variations in terms of frequency of new-onset MAU between the HR quartiles in the placebo group (*P* = 0.8253 for trend; Figure 3A). Incidence of MAU was high for all HRs, with approximately 10% of patients experiencing onset of the condition during the double-blind period of the trial. For the olmesartan group, however, there was a clear trend toward a lower incidence of MAU in the lower HR quartiles (*P* = 0.002 for trend). In the highest quartile, 10.5% of patients presented with MAU (similar to the placebo proportion), while the value for the lowest quartile was almost halved at 5.3%. Similar frequency distributions were found when patients were categorized according to last assessment or mean HR (Figure 3B, C). However, in these cases, the patients in the lowest HR quartile who were receiving the placebo experienced a slightly lower incidence of new-onset MAU in comparison to the other quartiles. The trend toward lower frequency of the condition in patients with a lower HR was maintained in the olmesartan group, independent of which measurements were used for defining the quartiles.

### Risk of Microalbuminuria According to Heart Rate and Treatment Assignment (Mean SBP Adjusted)

Patients in the lowest 3 quartiles of baseline HR were found to have a higher risk of new-onset MAU if they were receiving the placebo rather than olmesartan (Table 2). This was particularly evident when comparing incidence between the patients in the lowest quartile (OR: 1.92; 95% CI: 1.20–3.08). For the highest HR quartile, there was a little difference between the treatment and placebo arms (OR: 0.94; 95% CI: 0.63–1.40). After adjustment for mean SBP, the significant difference in risk of MAU between the 2 arms in the lowest HR quartile was maintained (OR: 1.75; 95% CI: 1.09–2.82).

**TABLE 2:**
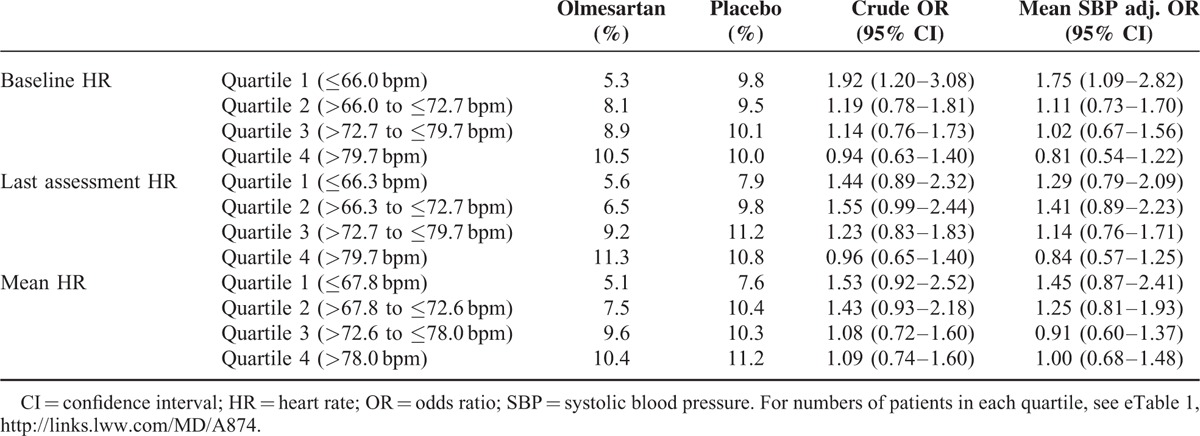
Frequency of New-onset Microalbuminuria According to Heart Rate by Treatment Group Both Crude and Adjusted for Mean SBP

Differences between the 2 arms were also found when last assessment HR was used to define the quartiles. For patients in the lowest HR quartile, there was a slightly but nonsignificantly higher risk of MAU in the placebo group (OR: 1.44; 95% CI: 0.89–2.32). On adjustment for mean SBP, the difference was reduced (OR: 1.29; 95% CI: 0.79–2.09). Similar values were found when mean HR was used for the division into quartiles.

## DISCUSSION

This subanalysis of the ROADMAP study demonstrated that for patients with type 2 diabetes, a lower HR was associated with a reduced risk of developing MAU. This was true more so for the olmesartan than for the placebo arm of the study.

### Association of Heart Rate and Microalbuminuria

When grouping the patients according to HR, patients in the lowest HR quartile were found to be at a lower risk of MAU than those in the highest quartile. This was independent of whether baseline HR, last assessment HR, or mean HR was used for defining the quartiles. These results are in agreement with data previously reported by Böhm et al, who showed a trend toward increasing MAU with increasing HR in patients with hypertension and/or cardiovascular disease.^14,22,23^ Similarly, Hillis et al^15^ noted an increasing risk of microvascular complications, including nephropathy, with increasing HR in patients with type 2 diabetes. In contrast, Pfister et al^24^ found no association between HR and prevalence of MAU in patients with type 2 diabetes and cardiovascular disease. However, MAU was much more common in this latter study, with 38.1% of patients developing the condition during the 2.9 years (average) of follow-up. It is possible that any link between HR and incidence of MAU was masked by the effect of the already pre-existing cardiovascular disease in all included patients. One strength of our study is that, in contrast to previously published data, the relationship between low HR with new onset of MAU was established in a population with lower cardiovascular risk.

In our study, the trend toward a lower risk of MAU with lower HR was upheld when the data were adjusted for mean SBP. This indicates that a lower HR is an independent predictor for a lower risk of new-onset MAU in patients with type 2 diabetes, thereby pointing to a specific protective effect of low HR on new-onset MAU.

### Association of Heart Rate and Microalbuminuria Considering Treatment Assignment

For patients assigned to the olmesartan arm of the study, there was a clear trend toward a lower frequency of new-onset MAU for patients in the lower HR quartiles. The incidence of MAU in the patients in the highest HR quartile was almost double than found for the lowest. In contrast, patients receiving the placebo demonstrated frequencies of new-onset MAU that were largely independent of HR quartile, and were similar to the rate found for olmesartan-treated patients in the highest HR quartile. Previously, it has been shown that a decrease in blood pressure results in a lower risk of albuminuria, and the question arose of whether olmesartan treatment provided protection against this microvascular complication solely through decreasing SBP.^25^ However, there are studies that have demonstrated a reno-protective effect of ARBs, which is independent of blood pressure,^21,26,27^ and according to our data, the observed trend toward a lower HR giving a lower risk of MAU was still apparent after adjusting for mean SBP. Furthermore, similar proportions of patients in the olmesartan and placebo arms of the study achieved the target blood pressure of <130/80 mm Hg during follow-up.^21^

Furthermore, it has to be noted that both the olmesartan and placebo pills were given on a background treatment that included beta-blockers (56.2%), calcium channel blockers (61.7%), and diuretics (51.3%). In each of these cases, more other drugs were used in the placebo group than in the olmesartan group because ROADMAP had a design aiming to control for the effects of blood pressure on outcomes (see Table 1). Sticking with the example of beta-blockers (58.4% in the placebo and 54.1% in the olmesartan group), it needs to be acknowledged that it will have reduced HR and has most likely affected the incidence of MAU, as seen for example for Atenolol in the LIFE trial.^28^

The greater association between olmesartan treatment and MAU found in the lower HR quartiles in comparison to the higher indicates that the effect of the drug may have been masked in the patients with a high HR. This observation merits further analysis.

### Limitations

One limitation of the present analysis is that it does not take into account a number of other potentially confounding factors such as diabetes duration and glycemic control. Furthermore, incidence of MAU was not directly correlated with changes in SBP during the double-blind period. However, the majority of patients in both the placebo and olmesartan groups achieved the target blood pressure of <130/80 mm Hg. A further potential limitation is the (low) number of missing values, for example, for HR at baseline (2 missings). We do not believe that this represents a serious limitation given the underlying randomized, controlled trial design that usually shows a data completeness far more than achievable in observational studies or databank analyses. Finally, this is an explorative subgroup analysis without randomized patient assignment.

## CONCLUSION

In patients with type 2 diabetes, a low HR was associated with a lower risk of developing MAU. This occurrence was particularly significant for patients being treated with olmesartan medoxomil. At higher HRs, the reduction in risk of MAU bestowed by the olmesartan appeared to be overcome by the effect of HR. The data demonstrate the potential benefits of reducing the HR of patients with type 2 diabetes and indicate that olmesartan could in particular reduce the risk of MAU in patients with low HR.

## Supplementary Material

Supplemental Digital Content
