# Supplementary material for: The Effect of Resting Heart Rate on the New Onset of Microalbuminuria in Patients With Type 2 Diabetes: A Subanalysis of the ROADMAP Study
Source: Medicine (Baltimore). 2016 Apr 18;95(15):e3122. doi: 10.1097/MD.0000000000003122 (PMC4839795; doi:10.1097/MD.0000000000003122)
Supplement: Supplemental Digital Content [file medi-95-e3122-s001.doc]

eTable 1: Assessment categories and quartile definition

|  | **Quartile 1** | **Quartile 2** | **Quartile 3** | **Quartile 4** |
| --- | --- | --- | --- | --- |
| **Baseline HR**  (N = 4,297)* | ≤66.0 bpm  (N = 1,100) | >66.0 to ≤72.7 bpm  (N = 1,092) | >72.7 to ≤79.7 bpm  (N = 1,045) | >79.7 bpm  (N = 1,060) |
| **Last assessment HR**  (N = 4,299) | ≤66.3 bpm  (N = 1,108) | >66.3 to ≤72.7 bpm  (N = 1,065) | >72.7 to ≤79.7 bpm  (N = 1,066) | >79.7 bpm  (N = 1,060) |
| **Mean HR**  (N = 4,299) | ≤67.8 bpm  (N = 1,075) | >67.8 to ≤72.6 bpm  (N = 1,076) | >72.6 to ≤78.0 bpm  (N = 1,075) | >78.0 bpm  (N = 1,073) |

*Legend: *missing values for 2 patients, 1 in the olmesartan and 1 in the placebo treated patient group*
